# Chernobyl: Relationship between the Number of Missing Newborn Boys and the Level of Radiation in the Czech Regions

**DOI:** 10.1289/ehp.10779

**Published:** 2007-09-14

**Authors:** Miroslav Peterka, Renata Peterková, Zbyněk Likovský

**Affiliations:** Department of Teratology, Institute of Experimental Medicine, Academy of Sciences CR v.v.i., Videnska, Prague, Czech Republic

**Keywords:** atomic power station, birth seasonality, ecologic catastrophe, newborn sex ratio, pregnancy outcome, radiation, radioiodine, raining, spontaneous abortion

## Abstract

**Background:**

The number of newborn boys was higher than that of girls in the Czech Republic each month from 1950 to 2005. The only exception was November 1986, when the number of newborn boys was significantly reduced. This has been explained by a selective negative impact of the Chernobyl accident in April 1986 on male fetuses during the third month of their prenatal development.

**Objectives:**

The first and most radioactive cloud passed over the Czech Republic during 30 April–1 May 1986. Concurrent rainfall multiplied the radioactivity by up to > 10,000-fold in specific regions. We verified a hypothesis that the decrease in the male birth fraction in November 1986 correlated with the level of radiation in eight Czech regions after the Chernobyl disaster.

**Results:**

We found a relationship between the level of radiation and the decrease in the number of newborn boys. The number of newborn boys was decreased in the six eastern regions where the radiation was strongly increased due to rain that accompanied the radioactive cloud. In contrast, the number of newborn boys was not reduced in the two western regions where the radioactivity was markedly lower.

**Conclusions:**

A negative impact of radiation on the prenatal population was manifested as a selective loss of newborn boys in November 1986. This loss correlated with level of radioactivity. The ^131^I probably played the most important role because of its up-take during primary saturation of fetal thyroid by iodine, which accompanies the onset of the gland function in 3-month-old fetuses.

The effect of relatively low doses of radiation from Chernobyl on pregnancy outcome is still under discussion in Europe. After the Chernobyl accident (26 April 1986), an increase in spontaneous abortions was reported not only in the adjacent area (Gavyliuk et al. 1992; Karakashian et al. 1997) but also in Finland (Auvinen et al. 2001) and Norway (Irgens et al. 1991; Ulstein et al. 1990). In Germany, perinatal mortality and trisomy 21 increased (Scherb et al. 2000; Sperling et al. 1994). Ericson and Kallen (1994) reported a slight excess of Down syndrome and childhood leukemia in Sweden among those who were *in utero* at the time of the accident. However, these authors did not exclude that the observed excess could be a random or temporary phenomenon. Increased incidence of thyroid diseases have been found in children in Hungary (Lukacs et al. 1997). In the Czech Republic, the data point to a significantly increased incidence of thyroid cancer (Murbeth et al. 2004).

We have shown that the impact of the Chernobyl disaster might also be manifested by a decrease in the male birth fraction in the Czech Republic in November 1986. There was a greater number of newborn boys (51.42%) than girls (48.58%) by 342.1 (2.83%), on average, in 599 of 600 months during the last 50 years. The only exception was November 1986, when the sex ratio reversed: Significantly fewer boys were born (49.35%) than girls (50.65%). We have estimated the number of missing boys to be 467 (Peterka et al. 2004).

Male fetuses are more vulnerable to prenatal damage by environmental stress (Nicolich et al. 2000). Thus, the long-standing higher male birth fraction is considered a sensitive indicator of stability and health in human reproduction (Campbell 2001; Davis et al. 1998; Vartiainen et al. 1999). Accordingly, a decrease in the male birth fraction has been reported after prenatal exposure to environmental chemical factors (Nicolich et al. 2000). In examining the cause of the exceptional reverse in the sex ratio in November 1986, we found that no full-area exposure to a harmful environmental factor occurred during 1986 other than the irradiation from the Chernobyl explosion. Therefore, the selective decrease in the newborn male fraction in November 1986 has been explained by a greater negative impact of radiation on male fetuses than on female fetuses. These fetuses passed the third month of prenatal development during the time of increased irradiation at the end April and beginning of May 1986 (Peterka et al. 2004).

Rainfall can strongly increase radiation on the ground (Pietrzak-Flis et al. 2003; Rosner et al. 1990; Stepanenko et al. 2002). The most radioactive cloud, accompanied by strong rain, passed above the eastern part of the Czech Republic on 30 April and 1 May 1986 (Kunz 1986). An increase in radioactivity from Chernobyl due to rainfall (Figure 1) can also be shown by Czech official data (Kunz 1986). The aim of the present study was to verify the hypothesis that the decrease in the male birth fraction in November 1986 correlated with levels of radiation after the Chernobyl disaster. The analysis performed in all eight Czech regions confirmed this hypothesis.

## Materials and Methods

We used official national demographic data registered by the Czech Statistical Office in Prague during 1950–2006. We first analyzed the monthly birth fractions of boys and girls (during 1950–1999) and the numbers of spontaneous abortions (during 1970–1999) for the whole Czech Republic. We then evaluated the numbers of newborn boys and girls for each of the eight Czech regions in each month during 1986 (Table 1).

We calculated the mean numbers of newborn boys and girls and the mean difference in their numbers (number of boys minus number of girls) for 11 months of 1986 (all months except November) and then for November alone.

To determine the number of missing newborn boys in November, we compared the difference between the numbers of newborn boys and girls in November 1986 with the mean difference between the numbers of newborn boys and girls during the rest of the year. The number of missing male births was then expressed as a percentage of the overall male birth rate in November 1986 in each of the individual regions. This method allowed the comparison of regions that have different birth rates (Table 1).

We assumed that the children born in November 1986 were at 8–12 weeks of gestation at the time of the highest radioactive fallout at the end of April and beginning of May 1986 (Peterka et al. 2004). We compared the data on birth rate in the Czech regions with the official data on radioactive fallout (represented by cesium-137) and rainfall intensity (in millimeters per day) measured from 0700 hours on 30 April 1986 until 0700 hours on 1 May 1986 (Kunz 1986). A correlation with personal data requiring special examination (e.g., fetal thyroid doses) was technically impossible with regard to the analyzed numbers of newborns in 1986 (> 130,000 babies).

Statistical significance of the number of newborn boys and girls was tested using the chi-square test and confidence interval of the mean (Dixon and Massey 1969).

## Results

### Seasonal variation in annual birth rates of girls and boys

We found two trends in the monthly birth rates during each year. First, in each year from 1950 to 1999, the birth rate gradually increased from December to reach its annual maximum in April. Then it gradually fell down to its annual minimum in November, with only one small increase in value in September (corresponding to fertilization in late December). Compared with the yearly mean birth rate and its confidence interval, we found a statistically significant baby boom in March, April, May, and June, and a significant decrease in October, November, and December.

The second trend was that more boys than girls were born each month (Figure 2); the only exception was November 1986, more girls were born (Figure 3).

### Seasonal variation in the number of spontaneous abortions

The number of spontaneous abortions showed regular changes each year from 1970 to 1999. For each year, the lowest numbers occurred in spring (April, May, June), and the highest numbers were recorded in winter (January, February) and in autumn (September, October). We did not find a marked increase in spontaneous abortions during 1986 (Figure 4).

### Radioactive cloud passage, rainfall, and radiation deposit

The radioactivity resulted from passage of the first radioactive cloud and was dramatically increased at specific places due to rainfall. The first and most radioactive cloud crossed the eastern part of the Czech Republic on 30 April 1986 (Kunz 1986) (Figure 5A). The most intense rain was recorded in the region of North Moravia from 30 April to1 May (Figure 5A).

The radioactivity deposit was irregularly distributed on the territory of the Czech Republic. The level of radiation deposit represented by ^137^Cs reflected well the crossing passage of the radioactive cloud and rainfall intensity (Figure 5B). The highest levels of radiation were recorded in all regions of the country except North and West Bohemia.

### Numbers of newborns in different Czech regions

In November 1986, the number of newborns ranged from 312 to 948 for boys and from 333 to 1,078 for girls in the individual Czech regions. During the remainder of 1986, the mean monthly numbers ranged from 391.4 to 1181.3 for boys and from 368.1 to 1112.5 for girls (Table 1). The mean monthly sex differences in the regions (from 23.0 to 68.8) documented that the number of newborn boys was always higher than that of girls (Table 1). The mean monthly numbers of newborns and the mean monthly sex differences (Table 1) suggest the expected numbers of newborn boys and girls and the expected difference in their numbers in November 1986 (Table 1). In six regions, the difference between the numbers of newborn boys and girls was lower than the mean difference in the rest of the year by 35–199 boys, which corresponded to 6–17% of the male birth rate (Table 1, Figure 5C).

Compared with the expected mean number of newborn boys, the most significant reduction in the male birth fraction in November 1986 was found in the regions of North Moravia and South Moravia, where 17% (199 boys; *p* < 0.01) and 14% (161 boys; *p* < 0.05) fewer boys were born, respectively (Figure 5C). In the regions of Prague, South Bohemia, East Bohemia, and Central Bohemia, 6% (35 boys), 11% (44 boys), 7% (47 boys), and 7% (41 boys) of male births were missing, respectively. We did not find a reduced male birth rate in North Bohemia or West Bohemia. In fact, more boys were born in these two regions, by 4% (27 boys) and 5% (25 boys) respectively, than predicted from the monthly mean for the rest of 1986.

### West–east gradient in the increasing number of missing newborn boys

In the Czech regions, the number of missing boys correlated with the extent of the area with a deposit of ^137^Cs at 5.5–30.0 kBq/m^2^ (Figure 5B). There was an apparent west–east gradient in the increasing number of missing boys: The maximum reduction occurred in the most eastern regions (North Moravia and South Moravia), with the greatest extent of the area with the highest radiation. The situation gradually improved in the west. The most westerly regions (West Bohemia and North Bohemia) showed minimum radiation deposits and no reduction in the number of boys (compare Figure 5B and Figure 5C).

## Discussion

In the present study, we found a seasonal variation in annual birth rates of girls and boys (Figure 2) and in the rate of spontaneous abortions (Figure 4). The variation in birth rate has been related to the photoperiod or temperature (affecting hormonal concentrations, sperm quality, or sexual activity) and may also be influenced by sociodemographic factors (Bobak and Gjonca 2001). The variation in spontaneous abortions can be influenced by lunar periodicity (Valandro et al. 2004).

In each year during 1950–1999, the maximum monthly numbers of newborn boys and girls were always in April, with the minimum in November. However, in each month (including November), more boys were born than girls (Figure 2). The reason that more boys are regularly born than girls has not yet been explained. The newborn sex ratio is determined during two steps. First, the primary sex ratio is established during conception; it results from the relative number and fertilizing capability of sperm bearing X or Y chromosomes that reach the ovum in the female genital tract. Second, the original sex ratio is further modified to reflect the relative survival of males and females during the prenatal period. It is not known whether the male excess results from a higher fertilizing capability of spermatozoa bearing Y chromosomes or from a preferential loss of female conceptuses during later periods (Bonde and Wilcox 2007).

A higher male birth ratio is considered a sensitive indicator of health in human reproduction (Campbell 2001; Davis et al. 1998; Vartiainen et al. 1999). In the Czech Republic, a unique and significant decrease in the male birth ratio was found in November 1986 (Peterka et al. 2004). This is the only time a reverse in the sex ratio has been observed at the whole population level in the Czech Republic. The irradiation from the Chernobyl accident was the only suspected harmful environmental factor with full-area impact that could be taken into consideration.

A decrease in the male birth ratio can result either from a change in the primarily determined sex ratio during conception or from a decrease in survival of males during the prenatal period. A change in the newborn sex ratio has been reported after paternal exposure to a harmful environmental factor, which resulted in a change in the sex ratio during conception (e.g., Fukuda et al. 2002; James 1996, 2002). However, Bonde and Wilcox (2007) have impeached the employment of a change in the sex ratio as a “barometer of male reproductive health.” In any case, the decrease in the male birth ratio in November 1986 could not be explained by a change in the primary (determined at conception) sex ratio because of a negative effect of Chernobyl radiation on male reproductive health manifested as a decrease in fertilization capability of Y-bearing sperm. The conception and determination of sex of prospective November newborns occurred in February 1986, 8–12 weeks before the Chernobyl explosion. Therefore, based on the second step in determining sex ratio, the boys missing in November must have been either born prematurely or aborted.

The missing newborn boys in November 1986 were not born prematurely because the numbers of male births in September and October 1986 were not increased. Neither did the number of newborn girls increase in November 1986 (Peterka et al. 2004). Therefore, it could be expected that the decrease in the boy birth ratio resulted from a previous increase in spontaneous abortions.

We evaluated data from the official Czech statistical monitoring system. Compared with other years, we did not find a marked change in spontaneous abortions during 1986 (Figure 4). However, it is necessary to take into account the important limits of the monitoring of spontaneous abortions. First, the register includes only those spontaneous abortions that took place in a hospital and were obligatorily reported by physicians; spontaneous abortions that occurred outside of hospitals are not included in the register. Second, data on sex are not recorded in the register of spontaneous abortions. Moreover, sex can reliably be determined by morphologic examination as late as the fourth prenatal month.

Birth represents a well-defined termination point in the overwhelming majority of pregnancies. The entire sample of prospective newborns follows a similar schedule, being born at a similar time. This is why the newborn data are robust and reliable. In contrast, there is no predictable fixed term for a spontaneous abortion, so we cannot expect that the population of fetuses damaged during the third month of prenatal development (i.e., late April through early May 1986) (Peterka et al. 2004) would suddenly be aborted, thereby giving rise to a significant peak on the annual curve of abortions. Therefore, significant spontaneous abortions of males or females could be indirectly detected as a change in the newborn sex ratio.

### Missing newborn boys in November 1986 and radioactivity levels

**Figure 1:**
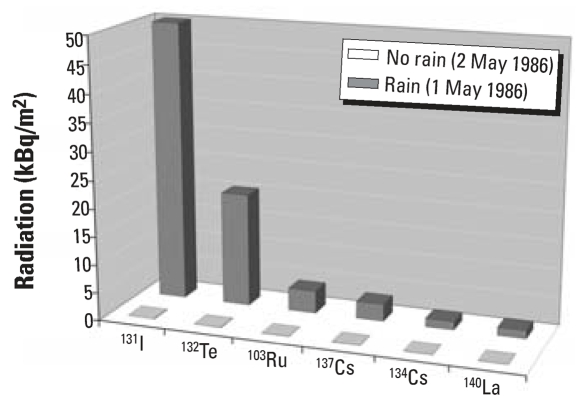
Levels of radioactive fallout in the center of the East Bohemia region of the Czech Republic. Six radionuclides (iodine-131, tellurium-132, ruthenium-103, cesium-137, cesium-134, and lanthanum-140) were measured at 0700 hours on 1 May (during rainfall) and on 2 May (with no rain). Data from Kunz (1986).

**Figure 2:**
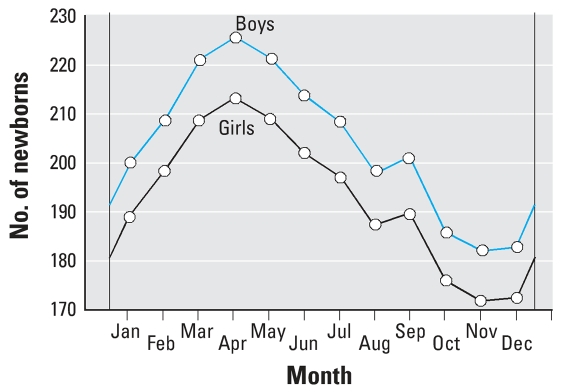
Seasonal variation (mean number per month) in the annual rates of living newborn boys and girls in the Czech Republic calculated from 1950–1999 data. To eliminate the problem arising from the varying number of days in a month (e.g., 31 days in January, 28 or 29 in February) for a total of 600 months, we calculated the mean number of newborns per day; we used that number to calculate a mean value for each month in a year.

**Figure 3:**
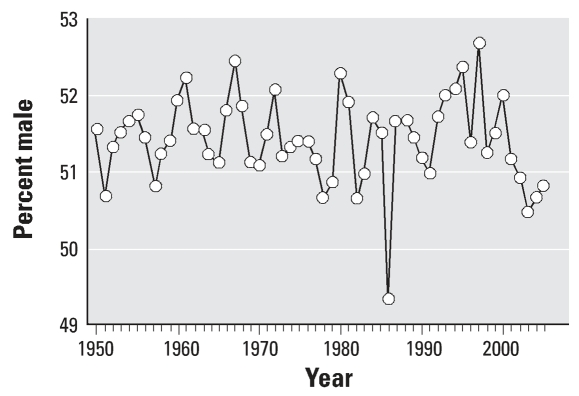
The percentage of boys among infants born in the Czech Republic in each November during 1950–2005. Only in November 1986 is the value < 50%, indicating that fewer boys were born than girls.

**Figure 4:**
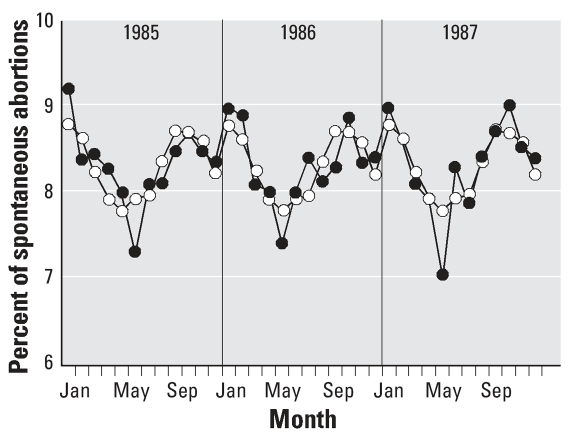
Seasonal variation in the annual percentage of spontaneous abortions officially registered in the Czech Republic. Values shown are percentage per month; total spontaneous abortions per year = 100%. Black circles indicate the percentage of spontaneous abortions calculated for each month during 1985, 1986, or 1987. Open circles indicate the mean annual rhythm calculated from data on spontaneous abortions in 1970–1999.

In the regions affected by the passage of the radioactive cloud accompanied by rain, the male birth ratio was much lower than in the regions that did not experience rainfall. The most affected area was the eastern part of the Czech Republic (North and South Moravia). In the western Czech Republic, the number of missing boys gradually decreased. At places where the strong radioactive cloud passed and rain simultaneously fell, the radioactivity of all measured radionuclides increased suddenly (Kunz 1986) (Figure 1). A similar increase in the deposit of radioactivity by rainfall during 26 April–6 May has been reported for Western Europe (Clarke 1986). There, a number of rainstorms increased the deposition of radioactive material > 100-fold.

The radiation-induced effects during the prenatal period are dependent upon the radiation dose and the stage of development when radiation exposure occurs. The most important effects of exposure to radiation during the embryonic or fetal period are *a*) the death of the embryo or fetus; *b*) malformations, changes in growth rate, or other functional changes; *c*) mental retardation; and *d*) the induction of malignancies including leukemia (Ogris 1997). After the Chernobyl catastrophe, two of these radiation effects have been found in the Czech Republic: a significant increase in thyroid carcinomas (Murbeth et al. 2004) and a lethal effect on male fetuses, detected indirectly due to the transient change in the newborn sex ratio in November 1986 (Peterka et al. 2004).

### Increased thyroid cancer in the adult Czech population

From 1990, an additional significant increase in thyroid cancer was observed with an incidence of 2.6%/year in the Czech Republic (Murbeth et al. 2004). This phenomenon was independent of age but dependent on sex (females, 2.9%/year; males, 1.8%/year). Because the estimated minimum latency between radiation exposure and an increase in thyroid cancer at the population level is 4 years, Murbeth et al. (2004) related the increase in thyroid cancer in the Czech population dating from 1990 to the radiation emitted after the Chernobyl disaster. We find it extremely interesting that the incidence of thyroid cancer did not significantly increase in West Bohemia (Murbeth et al. 2004). In the present study, we analyzed another parameter of the impact of radioactivity at the population level with a similar conclusion: the male birth ratio was not changed (newborn boys were not missing) in West Bohemia. Both results support our explanation that the level of radiation due to the meteorologic situation (wind direction and rainfall) was lower in the western part of the Czech Republic.

The increase in thyroid cancer has been related to the accumulation of ^131^I (Murbeth et al. 2004). Under normal conditions, ^131^I does not occur at measurable levels. However, in the Czech Republic, the level of ^131^I was the highest among all measured radionuclides—50 kBq/m^2^ in some regions after rainfall on 1 May (Kunz 1986). The radioactive half-life of ^131^I is only 8 days; therefore, the released amount of radioactivity is much greater than that from, for example, cesium or strontium, whose radioactive half-lives are as long as 30 years. We propose that both the increase in thyroid cancer incidence (Murbeth et al. 2004) and the loss of male fetuses (Peterka et al. 2004) in the Czech Republic might have a common cause—radioiodine.

### Radioiodine, the most probable cause of the death of male fetuses

It is generally known that ^131^I is selectively accumulated in the thyroid gland postnatally and prenatally. The radioactivity of ^131^I has been measured after an intravenous injection of 5 μCi radioiodinated human serum albumin in the gonads of a pregnant woman and her fetus and in the fetal thyroid gland (Hibbard and Herbert 1960); the radioactivity was almost 1,000 times higher in the fetal thyroid gland than in the fetal and maternal gonads. Berkovski et al. (2003) summarized data on the evaluation of fetal doses resulting from maternal intakes of radioiodine and documented the ratio of committed equivalent doses to thyroid glands of fetus and mother in case of acute ingestion of radioiodine at different gestation stages (Figure 6). These data clearly show the critical, > 1,000-fold increase of ^131^I in fetal thyroid gland during the third prenatal month (Figure 6). A similar critical period (prenatal weeks 10–12) for accumulation of ^131^I by fetal thyroid has also been reported by Zanzonico (1997). This fits with data about the physiologic acute increase of iodine content in the thyroid gland during the third prenatal month, just before the thyroid starts to function in human fetuses. Such a sudden intake of iodine represents a very vulnerable breakpoint in case the ^131^I is available in place of normal iodine. The selective uptake of ^131^I results in a very high acute dose of radiation in the fetus; this strongly supports our findings about the loss of fetuses irradiated during the third month of development.

The third month of prenatal development also fits with the critical period of vulnerability of the developing fetal brain to radiation injury reported after the atomic bombings in Hiroshima and Nagasaki, Japan (Yamazaki and Schull 1990). The radiation effect has been explained by the impaired migration of immature neurons to the brain cortex over the course of the 8th–15th weeks of prenatal development. The use of radioactive iodine is absolutely contraindicated during pregnancy because it can cause fetal or neonatal death (Bishnoi and Sachmechi 1996; Halnan 1985).

After the Chernobyl disaster, thyroid doses of ^131^I came from intake via inhalation and ingestion (Drozdovitch et al. 2007). Rain drew down ^131^I from the radioactive cloud to the ground and increased ground-level radioactivity to 50 kBq/m^2^ (Figure 1). At that time point, inhalation probably represented the most important route of intake and mediated the acute effect of ^131^I before the radionuclides entered the food chain. The secondary intake from digestion (e.g., milk) was similar for everyone in our country because of food distribution by companies.

Drozdovitch et al. (2007) estimated thyroid doses of ^131^I in the Czech Republic after Chernobyl for adults (1.7 mGy) and for children 1 year of age (11.7 mGy). We can assume that the fetal doses were much higher. When the fetal thyroid is just becoming functional, its iodine intake is maximal. The critical period of structural maturation of the fetal thyroid gland and onset of its function is the third month of prenatal development. The gland starts to synthesize thyroid hormones, which requires the presence of iodine. Therefore, iodine is rapidly absorbed by the thyroid gland, including ^131^I (Costa et al. 1991; Fisher and Klein 1981; Zanzonico 1997). The fetal thyroid gland has a 10- to 50-fold greater (Berlin 2001; Green et al. 1971; Russell et al. 1957) or 1,000-fold greater (Berkovski et al. 2003) accumulation of ^131^I than does the maternal thyroid.

**Figure 5:**
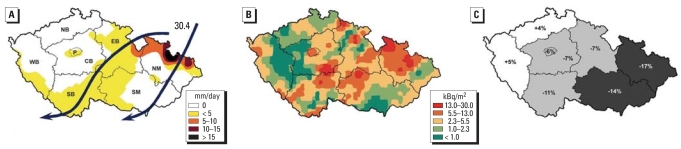
Schematic maps of the Czech Republic with delineated regions. Abbreviations: CB, Central Bohemia; EB, East Bohemia; NB, North Bohemia; NM, North Moravia; P, Prague; SB, South Bohemia; SM, South Moravia; WB, West Bohemia. (*A*) Passage of the first radioactive cloud over the country on 30 April (arrows) and the intensity of the rainfall measured from 0700 hours on 30 April until 0700 hours on 1 May 1986. (*B*) Distribution and level of radiation represented by ^137^Cs; Note that the highest radiation deposit was in North and South Moravia, which reflects the areas of rainfall at the time the radioactive cloud passed over; the lowest radiation deposit was recorded in the areas outside the passage of the radioactive cloud, in North and West Bohemia, where the rain was absent or minimal. (*C*) The percentage of missing boys in each region during November 1986.

**Figure 6:**
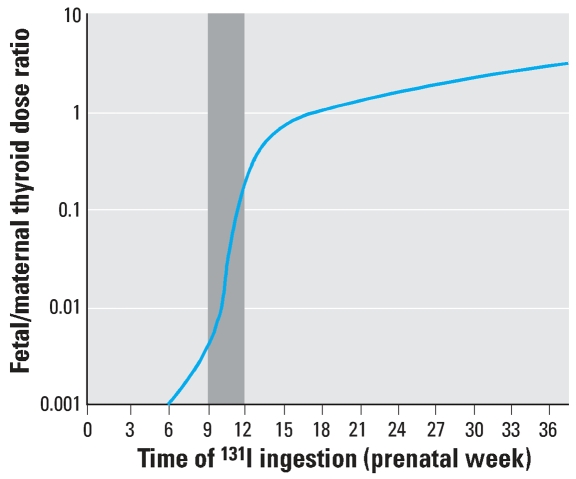
The fetal/maternal thyroid dose ratio in case of acute ingestion of ^131^I at different gestation stages (adapted from Berkovski et al. 2003). Note the enormous increase of the ^131^I content in the fetal thyroid during weeks 9–12 of prenatal development (gray bar).

Based on these data, we can estimate the fetal thyroid doses in the Czech Republic after the Chernobyl explosion: If the thyroid dose was 1.7 mGy in Czech adults (Drozdovitch et al. 2007), the fetal thyroid dose of ^131^I might even reach values of 17, 85, or 1,700 mGy. Moreover, it is necessary to take into account that the radioactive fallout of ^131^I was 255 times higher at the places affected by the radioactive cloud passage with rain than at the places without rain (Figure 5), and that the ^131^I was not the only radionuclide present in the fallout (Figure 1).

Uptake of ^131^I by the adult and fetal thyroid gland is enhanced by normal iodine deficit (in case the gland is not sufficiently saturated by iodine before exposure). The Czech Republic has a mild deficiency of iodine; thus the population suffers from a mild iodine deficiency. This undoubtedly contributed to the fact that Czech citizens suffered the greatest contamination of their thyroid glands by ^131^I of all 22 European countries after the Chernobyl accident (O’Hare et al. 1998).

We propose that the reduction in number of newborn boys after the Chernobyl explosion was due to the large release of ^131^I and other shorter-lived isotopes of iodine, which resulted in damage of the thyroid gland in fetuses and/or their mothers. Serious damage of the thyroid gland in 3-month-old fetuses at end of April–beginning of May 1986 by radioiodine might be one of the main reasons for their loss, which was detected as a decrease in the male birth ratio in November 1986.

### Protection against ^131^I

Due to the lack of normal iodine, the thyroid gland is especially susceptible to radioiodine. In contrast, a pharmacologic thyroid blockade by oral potassium iodine (50–100 mg in adults) can substantially reduce thyroid uptake of and irradiation by internalized radioiodine. Potassium iodide administered up 48 hr before ^131^I exposure can almost completely block thyroid uptake and therefore greatly reduce the ^131^I dose absorbed by thyroid (Zanzonico and Becker 2000). In Poland, potassium iodide was promptly distributed 3 days after the Chernobyl explosion. More than 95% of children and teenagers and 27% of adults (about 17 million people) took a protective single dose of potassium iodide on 29 April 1986 (Krajewski 1991; Nauman 1991; Nauman and Wolff 1993).

After the Chernobyl accident, the (former) Soviet Union and other countries adopted preventive programs aimed to overload the thyroid gland with stable iodine (potassium iodide) as soon as possible in case of another atomic power crash or a terrorist attack. Recently, the Israeli government decided to distribute potassium iodide tablets to citizens living near the two nuclear research centers in Israel (Kroizman-Sheiner et al. 2005). Special attention should be paid to the protection of young children (Smeesters et al. 1998) and pregnant women.

## Figures and Tables

**Table 1:** Calculation of the number and percentage of missing newborn boys in November 1986.

		Eleven months of 1986		November 1986 only			
Czech region	Sex	Monthly mean no. (± SE) of newborns	Monthly mean sex difference^a^	No. of newborns	Sex difference^a^	No. of missing boys^b^	Percent of missing boys^c^
North Moravia	M	1178.9 ± 15.0	+ 68.6	948	–130	–199	–17**
	F	1110.3 ± 12.0		1,078			
South Moravia	M	1181.3 ± 16.9	+ 68.8	930	–92	–161	–14*
	F	1112.5 ± 17.9		1,022			
South Bohemia	M	391.4 ± 13.2	+ 23.3	312	–21	–44	–11
	F	368.1 ± 9.9		333			
East Bohemia	M	700.4 ± 13.4	+ 48.2	561	+ 1	–47	–7
	F	652.2 ± 12.4		560			
Central Bohemia	M	605.6 ± 17.3	+ 36.1	496	–5	–41	–7
	F	569.5 ± 19.6		501			
Prague	M	565.8 ± 18.3	+ 39.8	448	+ 5	–35	–6
	F	526.0 ± 18.2		443			
West Bohemia	M	483.2 ± 28.7	+ 23.0	434	+ 48	+ 25	+ 4
	F	460.2 ± 21.9		386			
North Bohemia	M	691.3 ± 27.7	+ 41.9	633	+ 69	+ 27	+ 5
	F	649.4 ± 28.4		564			

Abbreviations: F, female; M, male.

a Number of newborn males – number of newborn females.

b Sex difference in November 1986 – the monthly mean sex difference for 11 months.

c No. of missing boys ÷ monthly mean no. of newborn males for 11 months.

* *p* < 0.05.

** *p* < 0.01.
